# Complete Genome Sequences of Five *Chrysodeixis chalcites* Nucleopolyhedrovirus Genotypes from a Canary Islands Isolate

**DOI:** 10.1128/genomeA.00873-13

**Published:** 2013-10-24

**Authors:** Alexandra Bernal, Trevor Williams, Delia Muñoz, Primitivo Caballero, Oihane Simón

**Affiliations:** Instituto de Agrobiotecnología, CSIC-Gobierno de Navarra, Mutilva Baja, Navarra, Spaina; Instituto de Ecología AC, Xalapa, Veracruz, Mexicob; Departamento Producción Agraria, Universidad Pública de Navarra, Campus Arrosadía, Pamplona, Navarra, Spainc

## Abstract

The *Chrysodeixis chalcites* single nucleopolyhedrovirus (ChchSNPV) infects and kills *C. chalcites* larvae, an important pest of banana crops in the Canary Islands. Five genotypes present in the most prevalent and widespread isolate in the Canary Islands were sequenced, providing genetic data relevant to the genotypic and phenotypic diversity of this virus.

## GENOME ANNOUNCEMENT

The *Chrysodeixis chalcites* single nucleopolyhedrovirus (ChchSNPV) (*Baculoviridae*: *Alphabaculovirus*) has great potential to be a complement or alternative to chemical control of its natural host, *C. chalcites* (*Lepidoptera*: *Noctuidae*), as occurs with many other baculovirus-host systems (1), particularly for banana protection in the Canary Islands, Spain. A Canarian isolate, ChchSNPV-TF1wt (ChchTF1), obtained from a *C. chalcites*-infected larva collected during a natural epizootic in banana crops, is the most prevalent and widespread isolate in the Canary Islands and displays the highest pathogenicity and virulence values compared to previously described strains from the Netherlands (2) and Spain (3, 4). This strain is composed of multiple genotypes, which have been cloned *in vitro* (5). The complete genome sequences of the three most abundant genotypes, namely, ChchTF1-A, ChchTF1-B, and ChchTF1-C, and the two scarcest ones, ChchTF1-G and ChchTF1-H, were determined by 454 sequencing, assembled with Newbler version 2.3 software, and checked in detail manually.

The genomes of *C. chalcites* ChchTF1-A, ChchTF1-B, ChchTF1-C, ChchTF1-G, and ChchTF1-H are 149,684, 149,080, 150,079, 149,039, and 149,624 bp long, respectively, very similar to the genome of the ChchSNPV type isolate, *C. chalcites* ChchSNPV-NL (149,622 bp) (GenBank accession no. AY864330), from the Netherlands. All five isolates have a 39% G+C content, also similar to that of ChchSNPV-NL (2). The unique ChchSNPV gene, open reading frame (ORF) 53, was not identified in the ChchTF1-A, ChchTF1-B, ChchTF1-C, or ChchTF1-H genome due to a single nucleotide mutation in the start codon (TGC). Hence, a total of 150 ORFs were predicted in ChchTF1-A, ChchTF1-B, ChchTF1-C, and ChchTF1-H, and 151 ORFs were predicted in the ChchTF1-G genome. Fifty-eight ORFs are 100% homologous in the six ChchSNPV genomes sequenced to date. The 62 genes conserved in other lepidopteran baculoviruses were all present (6). As was previously described for ChchSNPV-NL (2), no typical homologous regions (*hrs*) were identified in the ChchTF1 genotypes. A whole-genome sequence alignment between ChchTF1-A, ChchTF1-B, ChchTF1-C, ChchTF1-G, and ChchTF1-H and ChchSNPV-NL shows 98 to 99% homology at the nucleotide level. This analysis also demonstrated that variable genomic regions are located principally in the *hoar* and *bro-d* genes, which represent a major source of intraspecific variability among genotypes in many baculoviruses (7–9). Finally, phylogenetic analysis grouped the five Spanish genotypes and the Dutch genotype in three pairs of clusters: ChchSNPV-NL with ChchTF1-G, ChchTF1-A with ChchTF1-B, and ChchTF1-C with ChchTF1-H.

In all, our work will be helpful for further exploring the genetic diversity of this virus and the genes involved in insecticidal traits.

### Nucleotide sequence accession numbers.

The complete genome sequences of ChchSNPV-TF1-A, ChchSNPV-TF1-C, ChchSNPV-TF1-B, ChchSNPV-TF1-G, and ChchSNPV-TF1-H were submitted to GenBank under the accession no. JX535500, JX560539, JX560540, JX560541, and JX560542, respectively.
